# Development of top-dressing automation technology for sustainable shrimp aquaculture in India

**DOI:** 10.1007/s43621-021-00036-9

**Published:** 2021-05-24

**Authors:** Paulchamy Chellapandi

**Affiliations:** Industrial Systems Biology Lab, Department of Bioinformatics, School of Life Sciences, Bharathidasan University, Tiruchirappalli, Tamil Nadu 620024 India

**Keywords:** Shrimp, Probiotics, Automation, Disease control, Top-dressing, Immunity

## Abstract

Globally, the shrimp farming industry faces increasing challenges and pressure to reduce the broken shrimps and maintain a healthier pond environment. Shrimps lack an adaptive immune system to combat invading pathogens due to an imbalance in beneficial gut microbiota. The use of top-dressing agents like probiotics and pond optimizes is an alternative strategy to improve the innate immune system leading produce disease-free shrimp in international markets. The cost of top-dressing agents is accounted for 20% of the production cost and therefore, the development of top-dressing automation technology is important to maintain and improve the financial and environmental viability of shrimp sustainable farming. This perspective described several sensor-based aquaculture technologies for on-farm management systems but sustainability in the aquaculture industry is not yet achieved in practice. The present technology is a new invention to reduce labor and production costs required for reducing bacterial and organic loads in Biofloc shrimp cultures. Aquaculture automation system disperses the top-dressing agents to the shrimp ponds based on the signals received from microbial and environmental sensors. Continuous monitoring of shrimp growth, mortality, immune responses, diseases, and pond water quality parameters will fetch larger profits with additional savings on labor and production costs for sustainable shrimp aquaculture in India.

## Introduction

Shrimp farming is one of the most profitable and fastest-growing sectors in the aquaculture industry. The total global production of shrimp in 2017 was 4,267,500 metric tons of which Asian countries accounted for about 80.1% of production [1]. The Pacific white shrimp (*Litopenaeus vannamei*) is the most commercially important shrimp species in the world [2]. India is endowed with a long coastline and has a scope for large exploitation of marine wealth. In India, an estimated brackish water area suitable for shrimp culture is 11.91 lakh ha of which around 1.356 lakh ha area is currently under shrimp farming. *L. vannamei* is a benefited shrimp production in India. It is approved by the Coastal Aquaculture Authority of India in the States of Andhra Pradesh, Tamil Nadu, Maharashtra, Gujarat, Orissa, Goa, and Union Territories of Diu and Pondicherry. Andhra Pradesh has 974 km of coastline and 175,000 ha of brackish water. It produces more than half of the country’s farmed shrimp. Shrimp exports in India are increased by 16.21% (4,34,484 metric tons) during 2016–17. The Garret ranking and Rank Based Quotient analyses projected severe constraints in the shrimp aquaculture sector in India due to COVID-19 related lockdown [3]. However, a wide gap between the demand and supply increases global shrimp markets and overexploitation from coastal water [4]. About 76% of all farmed shrimp production is coming from the aquaculture sector. Value chain industries (aqua-feeds, equipment, chemicals, investments, and marketing, etc.,) are also contributed to the farmed shrimp-related economic growth. The fragmented market and global shrimp supply chains can be affected by the production of post-larvae shrimp by hatcheries [5].

Organic, traditional, and wastewater aquaculture methods are directly complementing the concepts of sustainability of shrimp culture [6]. Current environmental problems that plague the aquaculture industry are accomplished by organic aquaculture [7, 8]. Traditional aquaculture is effective in most rice-growing and fish-eating countries in Asia [9, 10]. The purposes of recycling organic wastewater for shrimp production and environmental protection are achieved with the wastewater aquaculture method [11]. Shrimp grow-out technology continues to be most extensive to semi-intensive and its efficiency should be improved through innovation and standardization of procedures. The grow-out shrimp are critically affected by overall operation success, risk of diseases, feeding control ratio, mortality, and low salinity [12]. Nurseries provide an opportunity for acclimation during the grow-out season. The sustainability of shrimp culture in the Biofloc system is very successful in practice [13–16]. However, increased energy costs, advanced technical skills, constant monitoring, and further research necessary to acclimatize Biofloc production. Various viral, bacterial and fungal diseases are also important challenges globally facing in Biofloc shrimp production worldwide [17, 18].

Commercially formulated high-energy feeds have been used for sustainable shrimp aquaculture in the prevention control of shrimp diseases and environmental crises [19]. In addition to the nutrients provided in the feed, farmers frequently top-dress their feed with vitamins, minerals, immune-stimulants, probiotics, and pond optimizers [20, 21]. Top-dressing agents are perceived by the farmers to be feed attractants and growth promoters [22, 23]. These agents have typically increased feed costs by US$39/ton in small-scale shrimp farming [24]. These agents can prevent the disease rather than the treatment of the disease in static or low water exchange systems. Farmers always aim to minimize overfeeding and usage of top-dressing agents to gain reasonable profits. There are several methods to be used for top-dressing applications. A traditional method requires constant hand labor for multiple top-dressing applications, which deteriorates the water and soil quality of the ponds. A mixture of feed and probiotics, adding probiotics in the feed ingredients and manual spraying top-dressing agents after feeding are traditional practices with the help of skilled laborers. A regular mild stirring of the pond bottom using iron chains to suspend the organic matter, followed by the addition of carbon sources can promote beneficial in-situ bacterial populations in culture ponds.

This practice results accumulation of an excess of ammonia and nitrite in the ponds. It can be solved by the addition of ammonia-oxidizing bacteria or nitrifying bacteria into the culture system upon the ammonia levels. Consequently, on-farm management gains major importance in the long-term sustainability of the aquaculture sector [25–28]. Therefore, there are no proper top-dressing automation methods for continuous monitoring and evaluation of shrimp growth and pond conditions. In this perspective, new top-dressing automation technology is suggested to reduce labor and production costs required for reducing bacterial and organic loads for sustainable shrimp aquaculture in India.

## Materials and methods

The PRISMA criteria were used in a methodical approach to this systematic review [29]. The information related to sensor-based automation methods and on-farm management was searched from the NCBI-PubMed databank and Google Scholars in February 2021. The following search parameters and MeSH terms “probiotics”, “sensor”, “Biofloc” “aquaculture”, “pond water”, “automation”, and “shrimp”. The inclusion criteria were the following: top-dressing agent, aquaculture automation system, on-farm management system, sustainable shrimp aquaculture, sensor-based automation, and pond water quality published in a peer-reviewed article and full-text publication. The exclusion criteria were the following: manual aquaculture methods, probiotics, shrimp production, and shrimp diseases. Most of the examined papers were published between 2000 and 2021. A total of 70 publications were evaluated for this systematic perspective by two independent experts. Online books were used for the definitions of aquaculture terms.

The regulatory concerns in environmental and economic sustainability issues affect shrimp farming in India [4]. A complex set of factors require multiple inputs and diverse perspectives in sustainable shrimp farming. Aqua-feeds, organic loads, chemical pollution, salinization of water, shrimp infections, and market failure are the nature of the environmental impacts associated with sustainable shrimp farming [30, 31]. These issues have also interacted with grow-out methods of shrimp aquaculture. Besides, biological community structures including bacteria, fungi, zooplankton, and phytoplankton have shown indirect effects on sustainable shrimp farming [32, 33]. White spot syndrome virus, infectious hypodermal and hematopoietic necrosis virus, monodon baculovirus, hepatopancreatic parvovirus, infectious myonecrosis virus, yellow head virus, and Taura syndrome virus are common viral diseases in shrimp aquaculture. Necrotizing hepatopancreatitis bacterium, acute hepatopancreatic necrosis disease, and enterocytozoon hepatopenaei are other microbial diseases affecting the growth of shrimp production. The PCR-based diagnoses of immune-response and probiotic competency-related genes are very important to know the health status of shrimp during the culture period (Table 1). Therefore, intensive production systems and sustainable practices that reduce the impact on local water quality and shrimp diseases can improve shrimp production in India.

**Table 1:** Details of PCR and RT-PCR primers for studying probiotics competency, biofilm-forming efficiency, Biofloc microbiota, and shrimp diseases

Target specifications	Primer	Primer sequence
Microbiota		
Bacteria	1114-F	CGGCAACGAGCGCAACCC
	1275-R	CCATTGTAGCACGTGTGTAGCC
Methanogens	McrA-F	TTCGGTGGATCDCARAGRGC
	McrA-R	GBARGTCGWAWCCGTAGAATCC
	Probe	ARGCACCKAACAMCATGGACACWGT
Lactobacillus	lacto-F	GAGGCAGCAGTAGGGAATCTTC
	lacto-R	GGCCAGTTACTACCTCTATCCTTCTTC
Immune-response and probiotic competency related genes		
F_1_F_0_-ATPase β-subunit	MGB-lacto	ATGGAGCAACGCCGC
	atpD-F	GCCAACCTGGTTCGTATGTG
	atpD-R	ACCACGTCGTCGATCTTACC
Bile salt hydrolase	bsh-F	ATGGGCGGACTAGGATTACC
	bsh-R	TGCCACTCTCTGTCTGCATC
Mucin binding protein	mub-F	GTAGTTACTCAGTGACGATCAATG
	mub-R	TAATTGTAAAGGTATAATCGGAGG
Mucus adhesion-promoting protein	mapA-F	TGGATTCTGCTTGAGGTAAG
	mapA-R	GACTAGTAATAACGCGACCG
Glyceraldehyde-3-phosphate dehydrogenase	Gadph-bac-F	ACTGAATTAGTTGCTATCTTAGAC
Nisin biosynthesis protein	nisB-F	GGGAGAGTTGCCGATGTTGT
	nisB-R	TAAAGCCACTCGTTAAAGGGCAAT
18 S rRNA (Shrimp)	18 S ShrRNA-F	GAGACGGCTACCACATCTAAG
	18 S ShrRNA-R	ATACGCTAGTGGAGCTGGA
β-Actin (Shrimp)	LvActin-F	CCACGAGACCACCTACAAC
	LvActin-B	AGCGAGGGCAGTGATTTC
Shrimp disease-coding genes		
White spot syndrome virus	WSSV-F	ATCATGGCTGCTTCACAGAC
	WSSV-R	GGCTGGAGAGGACAAGACAT
Infectious hypodermal and hematopoietic necrosis virus	IHHNV-F	TCCAACACTTAGTCAAAACCAA
	IHHNV-R	TGTCTGCTACGATGATTATCCA
Monodon baculovirus	MBV-F	CGATTCCATATCGGCCGAATA
	MBV-R	TTGGCATGCACTCCCTGAGAT
Hepatopancreatic parvovirus	HPV-F	GCATTACAAGAGCCAAGCAG
	HPV-R	ACACTCAGCCTCTACCTTGT
Infectious myonecrosis virus	IMNV-F	CGACGCTGCTAACCATACAA
	IMNV-R	ACTCGGCTGTTCGATCAAGT
Yellowhead virus	YHV-F	CCGCTAATTTCAAAAACTACG
	YHV-R	AAGGTGTTATGTCGAGGAAGT
Taura syndrome virus	TSV-F	AAGTAGACAGCCGCGCTT
	TSV-R	TCAATGAGAGCTTGGTCC
Necrotizing hepatopancreatitis bacterium	NHPB-F	CGTTGGAGGTTCGTCCTTCAGT
	NHPB-R	GCCATGAGGACCTGACATCATC
Acute hepatopancreatic necrosis disease	AHPND-F	ATGAGTAACAATATAAAACATGAAAC
	AHPND-R	ACGATTTCGACGTTCCCCAA
Enterocytozoon hepatopenaei	EHP-F	CAGCAGGCGCGAAAATTGTCCA
	EHP-R	AAGAGATATTGTATTGCGCTTGCTG

## On-farm management systems

Several on-farm management systems have been developed for sustainable shrimp aquaculture worldwide. An agricultural drip irrigation system for continuous water flow and a permanent supply of live food has been used for *L. vannamei* culture ponds [34]. TEMA software has been developed as a consultant for controlling pond water quality, productivity, and feeding [35]. A real-time automatic monitoring system has been used for continuous monitoring of pond water parameters [36]. ARM7 controller system has been modified for automatic detection and control of temperature, pH, and dissolved oxygen in aquaculture ponds [37]. The solar-powered automatic shrimp feeding system has been initiated to utilize a 10-hour timer to be set in intervals preferred by the user [38]. Auto Switch Aqua Feeder 1 has been designed for automation of feeding administration and substantially reducing the labor cost [39]. It helps to increase the shrimp growth rate and make shrimp healthier by living in high water quality. Aqua monitoring system using wireless sensor networks and IAR-Kick has been proto-typed to monitor and control an aquaculture system for semi-literate farmers to take suitable actions based on pond environmental information [40]. A patented prototype has been invented for a possible solution to automate and simplify the rearing of shrimps in the laboratory and the commercial ponds [41]. Hence, advances in automatic controlling systems and feeding algorithms support us to integrate environmental and microbial sensors for real-time monitoring of probiotics efficiency, pond water quality, and immune performance in shrimp farming.

## Sensor-based on-farm management systems

Commercial microbial and aquaculture sensors have been developed for online measurements of microbial growth and pond water quality parameters (Table 2). These aquaculture sensors are highly expensive but stable in hard environmental conditions for a long period of applications. Comparative analysis of sensor specifications indicated that environmental sensors available in Atlas Scientific are economically more suitable with desired quality for online applications in shrimp aquaculture. Ammonia sensor available in YSI Inc. is a low-cost one and appropriate to shrimp aquaculture applications. BACMON has been developed as a microbial sensor to detect the bacterial load in water continuously using automated batch sampling technology [42]. Hach’s has been developed a combination sensor for ammonium and nitrate and UVAS plus the reagent-free determination of the organic load in the ponds. Hach’s SC200 Universal Controller is the most versatile controller on the market and provides compatibility with the broadest range of sensors. AQ1 Sound Feeding System SF200 with complex filtering algorithms has been developed to analyze shrimp feeding sounds in aquaculture ponds using a hydrophone. AQ1 Adaptive© Feeding Algorithm has been implemented to control feed output to match the feed intensity precisely and eliminate feed waste using environmental sensors. FluoRAS sensor has been developed to monitor the accumulation of organic matter for optimizing recirculating aquaculture systems [43]. The online bacteria sensor has been developed based on 3D image recognition with algorithms considering 59 quantified image parameters for monitoring water quality [42]. A turbidity sensor has been designed based on the Beer–Lambert law for water quality monitoring in aquaculture ponds [44]. Low-cost environmental sensors have been designed, calibrated, and deployed to ensure their suitability for monitoring the water quality and fish behavior in aquaculture tanks during the feeding process [45]. The availability of environmental and microbial sensors and the deployment of wireless sensor networks could promote on-farm top-dressing practices [46].

**Table 2:** Commercial aquaculture sensors and their specifications

Parameters	YSI Inc.		Xylem Inc.		IN SITU Inc.		ATLAS Scientific	
	Model	Range	Model	Range	Model	Range	Model	Range
Temperature	006560	1–50 °C	4060R	1–40 °C	0063490	1–50 °C	V 1.2	1–99 °C
pH	577602	0–14	6589FR	0–14	0060000	0–14	V4.2	0–14
ORP	006565	± 300 mV	SensoLyt®SEA	± 2000 mV	0063470	± 1400 mV	V 2.2	± 2000 mV
Conductivity	599827	0–100 mS/cm	4119ASW	0–75 mS/cm	0063460	0–350 mS/cm	V 3.7	0.07–50 mS/cm
DO	006562	0–50 mg/L	FDO 700 IQ	0–20 mg/L	RDO®Blue	0–60 mg/L	V 4.6	0–100 mg/L
Turbidity	626901	0–4000 FNU	WQ 730	0-1000 FTU	0063480	0–4000 FNU	–	–
Ammonia	626906	0–200 mg/L	Tru Lab 1320	0.02–17,000 mg/L	0033700	0–10,000 mg/L	–	–
Nitrate	608090	0–10 mg/L	YSI MultiLab 4010-2	0.4–62000 mg/L	0033710	0–40,000 mg/L as N	–	–
Chlorophyll	599102-01	0–100 µg/L	YSI 6025	0–400 µg/L	0038900	0–1000 µg/L	–	–
BGA	626210	0–100 RFU	YSI 6031	0–100 RFU	0038930	0–100 RFU	–	–

## Top-dressing automation technology

Top-dressing agents (probiotics, ammonia-oxidizers, pond optimizers, etc.) can prevent the disease rather than the treatment of the disease and pond water quality until the culture period. Generally, top-dressing agents are very expensive to the production cost and have been applied to aquaculture ponds manually by a simple hand-throwing method. The automatic top-dressing system is a part of the on-farm management system in addition to automating feeding technology. It can automatically disperse top-dressing agents to the shrimp ponds based on the signals received from environmental and microbial sensors. It can automatically operate a solenoid valve to dispense the probiotics and pond optimizers from separate top-dressing tanks for managing pond water quality and making a favorable environment for healthier shrimp growth. It also operates an aerator to dispense air in the pond based on the concentration of dissolved oxygen.

## Experimental biofloc system

**Fig. 1:**
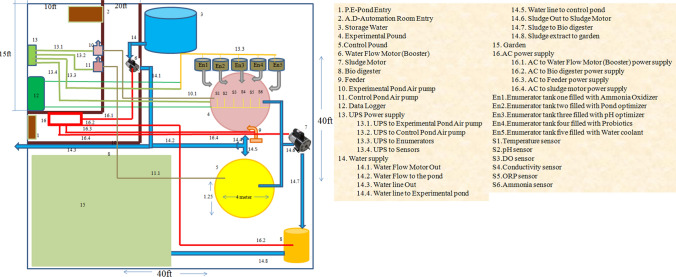
Schematic representation of aquaculture automation designing for Biofloc production of shrimp

**Fig. 2:**
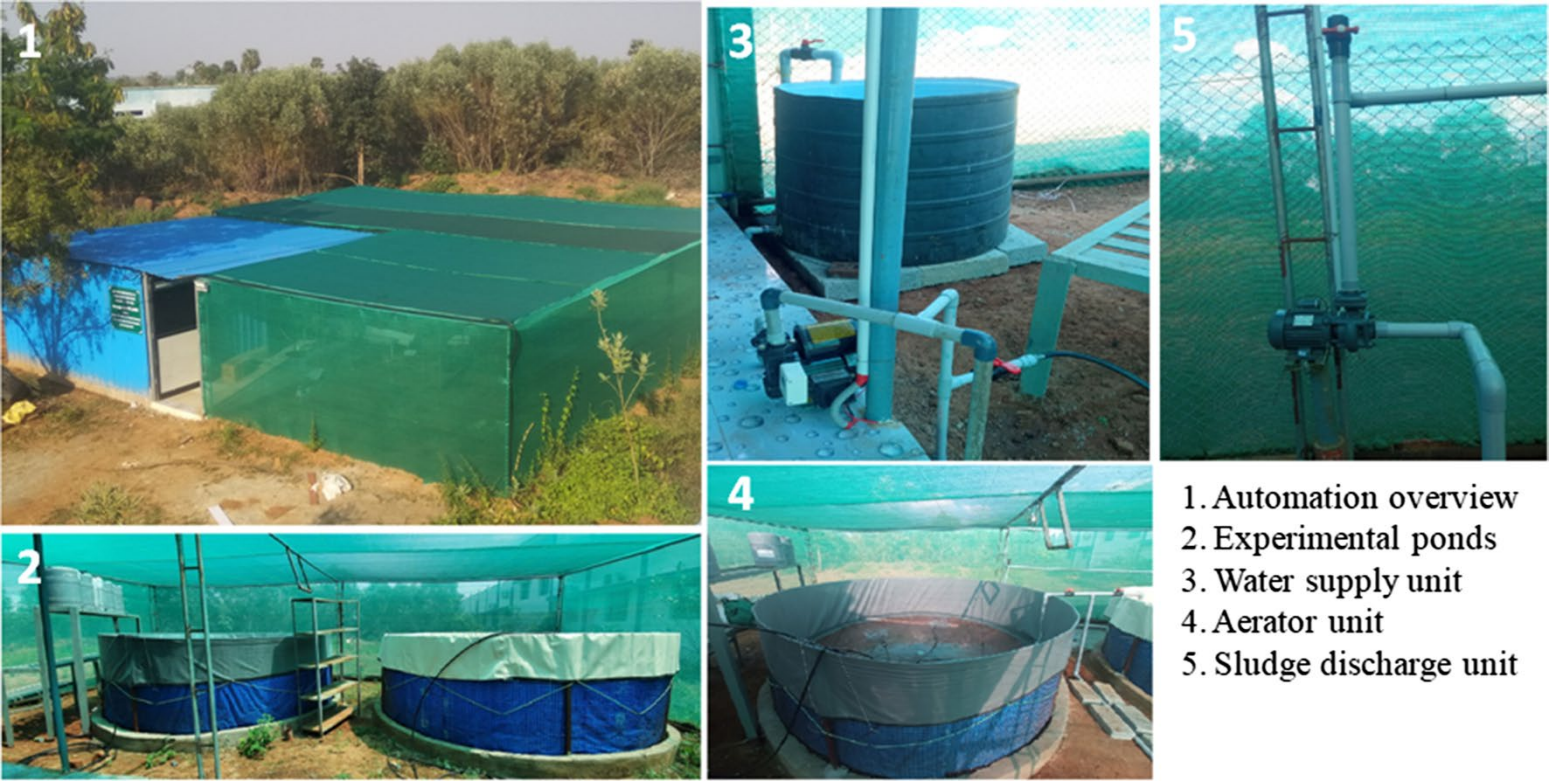
depicts the overview of experimental aquaculture automation system for Biofloc production of shrimp (Place: Bharathidasan University, Tiruchirappalli, India)

**Fig. 3:**
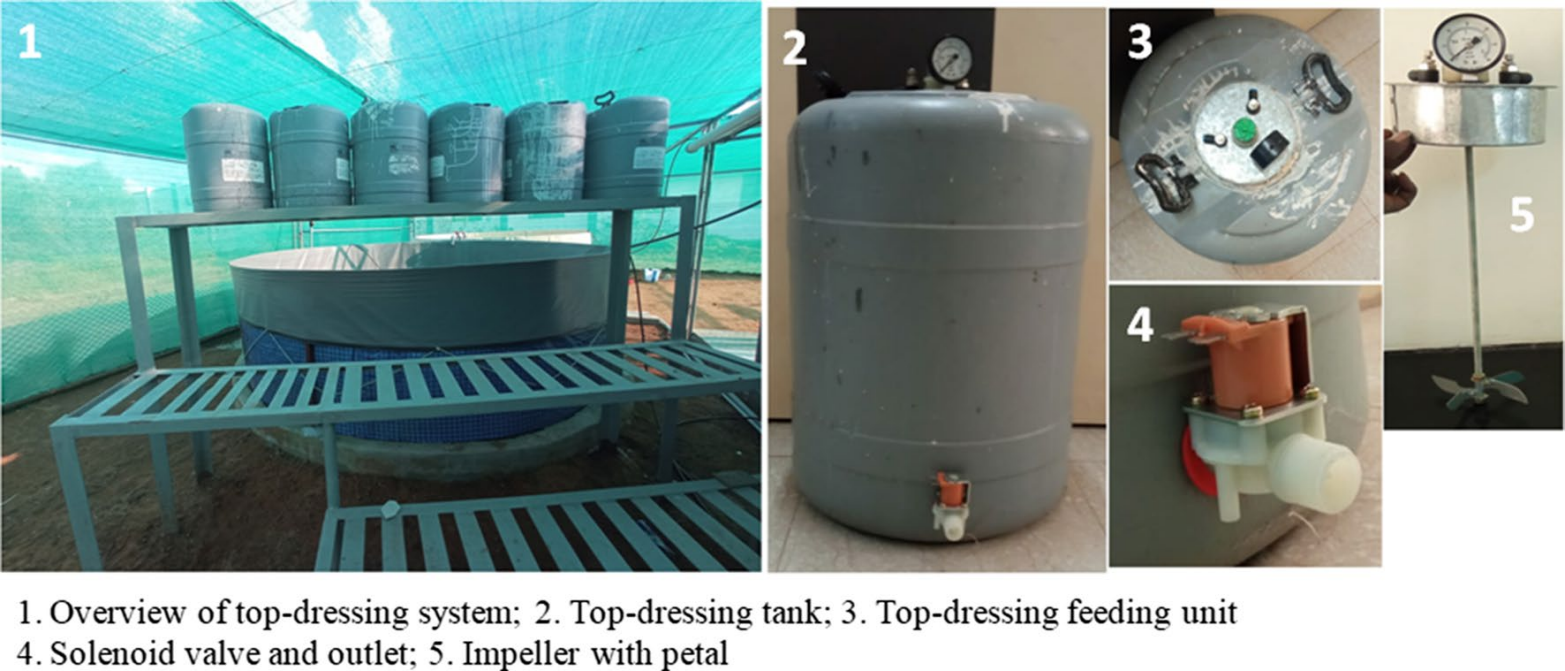
Top-dressing system with stirring and dispersion units to be used in an aquaculture automation system for Biofloc production of shrimp

The total volume of the proposed Biofloc pond is 12,000 L (4 m in diameter and 1.25 m in height) and the working volume is 10,000 L ((Figs. 1 and 2). Each pond is made up of tarpaulin material and covered with iron mesh. The aquaculture automation system is based on zero-waste water technology because the water supply is equivalent to discharged water. A pressure pump provides a 100 L water supply from the water sump to an experimental pond at a specific time. At the same time, the sludge pump discharges 100 L of excretory and feed waste products from the pond to 500 L anaerobic digester where those wastes are utilized as feedstock for biogas production. The digester effluent is drained off to the experimental garden for the cultivation of nursery plants. The total volume of the top-dressing tank (plastic) is 50 L and the working volume is 35 L (Fig. 3). It consists of an iron impeller that shaft is connected with an electric gear motor for stirring the top-dressing contents before dispersing to the pond. The outlet of this tank is connected with a solenoid valve for dispensing top-dressing agents. The iron lid of this tank has a provision of an inlet and outlet for filling the top-dressing agents.

## On-line measurement of water quality

**Fig. 4:**
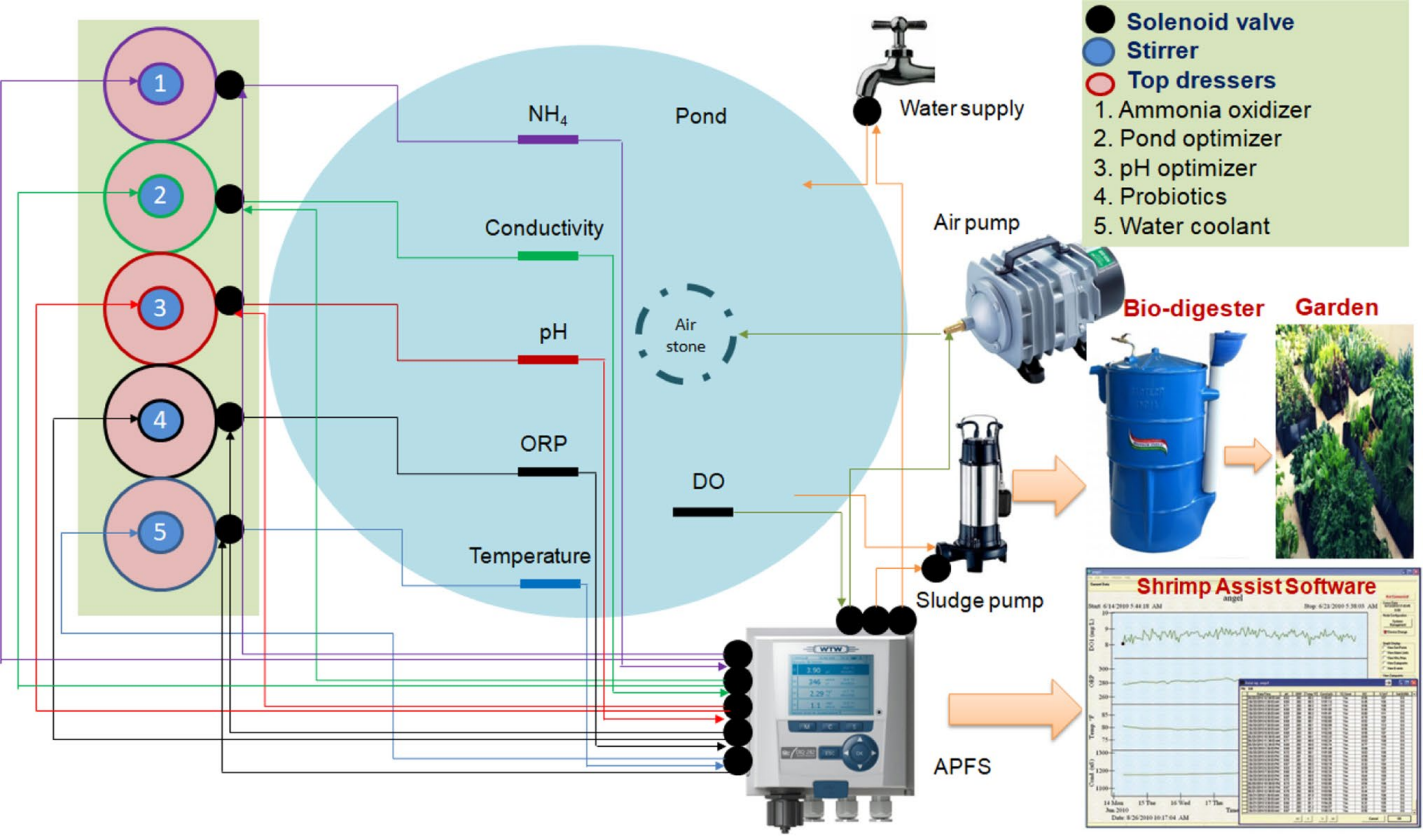
The proposed model for a top-dressing automation system for Biofloc production of shrimp

We should follow standard operation procedures for sustainable and eco-friendly Biofloc-based shrimp culture. All the physicochemical parameters should be maintained at an optimal level for better production of shrimp. The aquaculture automation system can continuously monitor the pond water quality in the Biofloc system during the culture period (Fig. 4). It can receive signals from sensors for temperature, pH, conductivity, dissolved oxygen, oxidation-reduction potential, and ammonia. Therefore, the relevant online monitoring sensors should be integrated with a controller to execute aquaculture automation systems in the Biofloc shrimp culture [13–16].

## Environmental sensors

Temperature is one of the most influential parameters in the pond system, which affects the metabolic rate of a living system, dissolved oxygen, and ammonia during the culture period. The optimum temperature is 28–30 °C for sustainable shrimp aquaculture [47]. The pH is to be maintained in the range between 6.8 and 8.0 due to ammonia–nitrate-nitrogen conversion processes in Biofloc [48]. Probiotic bacteria depend on suspended solids as a substrate for adhesion and as a source of energy from carbon in Biofloc systems [49]. To ensure efficient bacterial activity and a good system to control ammonia, total suspended solids should be maintained in the range of 250–450 ppm and organic load to be 5–15 mg L^− 1^ [50]. Dissolve oxygen should be maintained at 7–8 ppm to ensure the proper functioning of the system due to the oxygen demand of algae and bacteria in the Biofloc system [51]. Oxidation-Reduction Potential (ORP) sensors measure the oxidizing or reducing tendency of pond water and its optimal value to be 650–700 mV. In the optimal Biofloc production of shrimp, it should be maintained for aerobic bacteria (300–800 mV), facultative anaerobes (300 to − 100 mV), and anaerobic bacteria (− 700 to 200 mV) [52–54]. A conductivity sensor monitors the ability of a solution to conduct an electrical current. Conductivity is directly proportional to the concentrations of metal ions in the pond and therefore, it should be maintained as 2.7 dS m^− 1^ in the Biofloc system [55, 56].

The adjustment of the C:N ratio is the important factor for microbial growth in the Biofloc system. The optimum C:N ratio in an aquaculture system is 12–20:1. It has been adjusted by adding carbon sources and/or reducing protein percentage in feed [57]. Ammonium is a potential biomarker of an enzymatic by-product in the key physiological reactions of shrimp and microorganisms. It is considered to be a quality indicator of the Biofloc system. The recommended ammonia concentration in Biofloc culture is less than 1.2 and 6.5 ppm. The concentration of nitrite and nitrate should be in the range of 1 mg L^− 1^ and the range of 0.5–20 mg L^− 1^, respectively [58–62]. Hence, an ammonium sensor is suggested for the measurement of ammonium ions using an aquaculture automation system. It automatically disperses the ammonia-oxidizing bacteria or ammonia-oxidizing chemicals to the bottom of ponds based on ammonia and nitrite levels monitored by a combined ammonia and nitrite sensor.

## Microbial sensors

Multi-strain probiotic bacteria are commercially procured and formulated in a top-dressing tank containing formulated microbiological media supplements and pond water [63]. Bacterial culture is occasionally stirred by an electric impeller and cultured for 36–48 h at ambient temperature in a semi-continuous mode. Enriched probiotic culture is pumped to the ponds based on the signal receiving from the organic load/bacterial sensor through an automation controller. A top-dressing tank consisting of enriched ammonia-oxidizing archaea/bacteria is formulated in a defined ratio and diluted with pond water containing media ingredients [64–66]. Ammonia-oxidizing cultures are enumerated for 120 h at ambient temperature in a semi-continuous mode and then applied to the pond based on a signal received from the ammonia sensor.

## Automatic controlling system

An autonomous precision farming system is a control unit with the required customization at the hardware and firmware level for automation of top-dressing units in experimental ponds (Fig. 4). It is a user-friendly device that helps the farmers to plan and control the field activities. It monitors different parameters and informs the farmer through a graphical display unit. It also intelligently operates pumps/valves based on the data available from the field and the preloaded programs available in the automation system. The programs can be fed to the controller through the external memory interface. Auto, Manual, and Timer modes are in the controller system. The system integrates all feed-related information, such as the concentration of probiotics and pond optimizers, time of dispersal, and the interval between rations. These operations are automatically determined by the automation system on analyzing the sensor signals from the pond environment. All major units are designed farmer-friendly and assembled with technical experts. The field signals are the aquaculture sensors output that senses the various field parameters. As per the sensed signal thresholds, the autonomous precision farming system executes the control algorithm and generates the corresponding control signals to the field instruments.

## Sustainability in shrimp aquaculture

The proposed aquaculture technology is a sensor-based automation system that has options to set the frequency and quantity of multi-strain probiotics, pond optimizers, and continuous aeration. It can improve shrimp production and profits, and minimize environmental pollution from shrimp farming. It is also reliable to dispense more viable probiotics to the shrimp gut in the ponds because the commercial probiotics formulation is not ideally viable in the pond environment. It automatically dispenses the top-dressing agents from a radial sample range to allow effective coverage of the “feeding zone” in shrimp farms. It drives productivity by reducing the usage of top-dressing agents, increasing growth, minimizing environmental outputs, maximizing profits with minimal labor, and improving the consistency of size and flesh characteristics at harvest. It should be easy to maintain, long life in small-scale farms, and eliminate feed wastes by minimizing bacterial and organic load in the ponds. Nevertheless, continuous electricity and a relatively high level of technical expertise requirements would be costly to install and operate making them unsuitable for small-scale shrimp farmers.

## Socio-economic development

Global trends in antimicrobial use, antibiotics resistance, and health of shrimp gut microbiome and emerging diseases are major constraints in the shrimp industry today [67–69]. Moreover, the transfer of complex technical messages is problematic and requires continuous attention and extension as most farmers have relatively limited education [3]. Farmers must be adequately trained on new aquaculture technologies relevant to operations and on-farm top-dressing practices. Technical support should provide to the primary aquaculture societies and build capacity among small-scale farmers to reduce risks and to produce quality shrimp sustainably. The government should urge the researchers to partner in publicly funded research with the commercial probiotics manufacturing sector and aquaculture device manufacturing companies. Aquaculture engineering companies, agriculture, and state cooperative banks should provide financial support or subsidiary to implement that technology in the small-scale shrimp farmers. Aquaculture engineering intervention could be used to promote the development of better top-dressing technologies and allow research results to be more effectively transferred to farmers to improve feed utilization indices and reduce top-dressing costs [70].

## Conclusions

Top-dressing agents are used for the prevention and control of shrimp diseases and to improve growth yield in Biofloc systems. The cost of probiotics and pond optimizers is restricting the financial and environmental viability of shrimp aquaculture farming. Traditional methods could not help small-scale farmers to achieve sustainable aquaculture in India. Consequently, this perspective highlights the importance of a sensor-based top-dressing automation system for on-farm aquaculture management in India. The farmer-friendly controller automatically dispenses the top-dressing agents to ponds based on the signals received from each sensor. It gains a great interest in sustainable commercial aquaculture in a long period without any demand for major units. It will be a new aquaculture technology to reduce labor and production costs in Biofloc systems. Continuous monitoring of pond water parameters and microbial activity will reduce the labor and production costs for shrimp farming. Sustainable utilization of automatic aquaculture systems can thus lead to enormous social and economic benefits to the coastal regions of the country. The development of sustainable shrimp culture is possible only with some form of government intervention such as more efficient and environmentally friendly practices and awarding operating licenses. This sensor-based top-dressing automation technology will increase our scientific knowledge to achieving Goal-14 of the 2030 agenda for the development of sustainable aquaculture. Consequently, it will cut the cost of production, enhance the conservation and sustainable use of brackish water, and increase the economic benefits of developing countries in the future.

## References

[1] Hosain MA, Ullah K, Al Sayam MA, Mohiuddin K, Rahman E. Present status and future direction of Bangladeshi shrimp resources. Fish Aqua J. 2021;12:276.

[2] Zeng S, Huang Z, Hou D, Liu J, Weng S, He J. Composition, diversity and function of intestinal microbiota in pacific white shrimp (*Litopenaeus vannamei*) at different culture stages. Peer J. 2017;5:e3986. 29134144 10.7717/peerj.3986 PMC5678505

[3] Kumaran M, Geetha R, Antony J, Vasagam KPK, Anand PR, Ravisankar T, Angel JRJ, De D, Muralidhar M, Patil PK, Vijayan KK. Prospective impact of Corona virus disease (COVID-19) related lockdown on shrimp aquaculture sector in India—a sectoral assessment. Aquaculture. 2021;531:735922. 32939099 10.1016/j.aquaculture.2020.735922 PMC7484627

[4] Hossain MS, Uddin MJ, Fakhruddin ANM. Impacts of shrimp farming on the coastal environment of Bangladesh and approach for management. Rev Environ Sci Biotechnol. 2013;12:313–32.

[5] Quyen NTK, Hien HV, Khoi LND, Yagi N, Karia Lerøy Riple A. Quality management practices of intensive whiteleg shrimp (*Litopenaeus vannamei*) farming: a study of the Mekong Delta. Vietnam Sustainability. 2020;12:4520.

[6] Boyd CE, D’Abramo LR, Glencross BD, Huyben DC, Juarez LM, Lockwood GS, McNevin AA, Tacon AGJ, Teletchea F, Tomasso JR, Tucker CS, Valenti WC. Achieving sustainable aquaculture: historical and current perspectives and future needs and challenges. J World Aquaculture Soc. 2020;51:578–633.

[7] Martinez-Porchas M, Martinez-Cordova LR. World aquaculture: environmental impacts and troubleshooting alternatives. Sci World J. 2012;2012:389623. 10.1100/2012/389623 PMC3353277 22649291

[8] Islam M, Yasmin R. Impact of aquaculture and contemporary environmental issues in Bangladesh. Int J Fish Aquat Stud. 2017;5:100–7.

[9] Bostock J, McAndrew B, Richards R, et al. Aquaculture: global status and trends. Philos Trans R Soc Lond B Biol Sci. 2010;365:2897–912. 20713392 10.1098/rstb.2010.0170 PMC2935128

[10] Tezzo X, Bush SR, Oosterveer P, Belton B. Food system perspective on fisheries and aquaculture development in Asia. Agric Hum Values. 2021;38:73–90.

[11] Bluem V, Paris F. Aquatic modules for bioregenerative life support systems based on the C.E.B.A.S. biotechnology. Acta Astronaut. 2001;48:287–97. 11858270 10.1016/s0094-5765(01)00025-x

[12] Shinji J, Nohara S, Yagi N, Wilder M. Bio-economic analysis of super-intensive closed shrimp farming and improvement of management plans: a case study in Japan. Fish Sci. 2019;85:1055–65.

[13] Schock TB, Duke J, Goodson A, Weldon D, Brunson J, Leffler JW, Bearden DW. Evaluation of Pacific white shrimp (*Litopenaeus vannamei*) health during a superintensive aquaculture growout using NMR-based metabolomics. PLoS ONE. 2013;8:e59521. 23555690 10.1371/journal.pone.0059521 PMC3608720

[14] Santhana Kumar V, Pandey PK, Anand T, Bhuvaneswari GR, Dhinakaran A, Kumar S. Biofloc improves water, effluent quality and growth parameters of *Penaeus vannamei* in an intensive culture system. J Environ Manage. 2018;215:206–15. 29573671 10.1016/j.jenvman.2018.03.015

[15] Deng Y, Xu X, Yin X, Lu H, Chen G, Yu J, Ruan Y. Effect of stock density on the microbial community in biofloc water and Pacific white shrimp (*Litopenaeus vannamei*) gut microbiota. Appl Microbiol Biotechnol. 2019;103:4241–52. 30953119 10.1007/s00253-019-09773-4

[16] Manan H, Amin-Safwan A, Azman Kasan N, Ikhwanuddin M. Effects of biofloc application on survival rate, growth performance and specific growth rate of Pacific whiteleg shrimp, *Penaeus vannamei* culture in closed hatchery system. Pak J Biol Sci. 2020;23:1563–71. 33274888 10.3923/pjbs.2020.1563.1571

[17] Asche F, Anderson JL, Botta R, Kumar G, Abrahamsen EB, Nguyen LT, Valderrama D. The economics of shrimp disease. J Invertebr Pathol. 2020;21:107397. 10.1016/j.jip.2020.107397 32446865

[18] Kumar V, Wille M, Lourenço TM, Bossier P. Biofloc-based enhanced survival of *Litopenaeus vannamei* upon AHPND-causing *Vibrio parahaemolyticus* challenge is partially mediated by reduced expression of its virulence genes. Front Microbiol. 2020;11:1270. 32670225 10.3389/fmicb.2020.01270 PMC7326785

[19] Nguyen KAT, Nguyen TAT, Jolly C, Nguelifack BM. Economic efficiency of extensive and intensive shrimp production under conditions of disease and natural disaster risks in Khánh Hòa and Trà Vinh provinces. Vietnam Sustain. 2020;12:2140.

[20] Newaj-Fyzul A, Austin B. Probiotics, immunostimulants, plant products and oral vaccines, and their role as feed supplements in the control of bacterial fish diseases. J Fish Dis. 2015;38:937–55. 25287254 10.1111/jfd.12313

[21] Ray AK, Gopal C, Solanki HG, Ravisankar T, Patil PK. Effect of orally administered vibrio bacterin on immunity, survival and growth in tiger shrimp (*Penaeus monodon*) grow-out culture ponds. Lett Appl Microbiol. 2017;65:475–81. 28983933 10.1111/lam.12802

[22] Quiroz-Guzmán E, Vázquez-Juárez R, Luna-González A, Balcázar JL, Barajas-Sandoval DR, Martínez-Díaz SF. Administration of probiotics improves the brine shrimp production and prevents detrimental effects of pathogenic *Vibrio* species. Mar Biotechnol (NY). 2018;20:512–9. 29644500 10.1007/s10126-018-9822-8

[23] Wang YC, Hu SY, Chiu CS, Liu CH. Multiple-strain probiotics appear to be more effective in improving the growth performance and health status of white shrimp, *Litopenaeus vannamei*, than single probiotic strains. Fish Shellfish Immunol. 2019;84:1050–8. 30419396 10.1016/j.fsi.2018.11.017

[24] Ramaswamy UN, Mohan AB, Metian M. On-farm feed management practices for black tiger shrimp (*Penaeus monodon*) in India. In: On-farm feeding and feed management in aquaculture. Ed. Hasan MR, New MB. FAO Fisheries aquaculture technical paper. 2013;583:303–36.

[25] Bostock J, McAndrew B, Richards R, Jauncey K, Telfer T, Lorenzen K, Little D, Ross L, Handisyde N, Gatward I, Corner R. Aquaculture: global status and trends. Philos Trans R Soc Lond B Biol Sci. 2010;365:2897–912. 20713392 10.1098/rstb.2010.0170 PMC2935128

[26] Bentzon-Tilia M, Sonnenschein EC, Gram L. Monitoring and managing microbes in aquaculture towards a sustainable industry. Microb Biotechnol. 2016;9:576–84. 27452663 10.1111/1751-7915.12392 PMC4993175

[27] Ali H, Rahman MM, Rico A, Jaman A, Basak SK, Islam MM, Khan N, Keus HJ, Mohan CV. An assessment of health management practices and occupational health hazards in tiger shrimp (*Penaeus monodon*) and freshwater prawn (*Macrobrachium rosenbergii*) aquaculture in Bangladesh. Vet Anim Sci. 2018;5:10–9. 32734040 10.1016/j.vas.2018.01.002 PMC7386765

[28] Dhar AR, Uddin MT, Roy MK. Assessment of organic shrimp farming sustainability from economic and environmental viewpoints in Bangladesh. Environ Res. 2020;180:108879. 31706599 10.1016/j.envres.2019.108879

[29] Moher D, Liberati A, Tetzlaff J, Altman DG. Preferred reporting items for systematic reviews and meta-analyses: the PRISMA statement. PLoS Med. 2009;6:e1000097. 19621072 10.1371/journal.pmed.1000097 PMC2707599

[30] Páez-Osuna F. The environmental impact of shrimp aquaculture: causes, effects, and mitigating alternatives. Environ Manage. 2001;28:131–40. 11436996 10.1007/s002670010212

[31] Joffre OM, Klerkx L, Khoa TND. Aquaculture innovation system analysis of transition to sustainable intensification in shrimp farming. Agron Sustain Dev. 2018;38:34.

[32] Alfiansah YR, Peters S, Harder J, Hassenrück C, Gärdes A. Structure and co-occurrence patterns of bacterial communities associated with white faeces disease outbreaks in Pacific white-leg shrimp *Penaeus vannamei* aquaculture. Sci Rep. 2020;10:11980. 32686764 10.1038/s41598-020-68891-6 PMC7371890

[33] He Z, Pan L, Zhang M, Zhang M, Huang F, Gao S. Metagenomic comparison of structure and function of microbial community between water, effluent and shrimp intestine of higher place *Litopenaeus vannamei* ponds. J Appl Microbiol. 2020;129:243–55. 32043695 10.1111/jam.14610

[34] Ramirez JL, Avila S, Ibarra AM. Optimization of forage in two food-filtering organisms with the use of a continuous, low-food concentration, agricultural drip system. Aquacult Eng. 1999;20:175–89.

[35] Hernandez-Llamas A, Villarreal-Colmenares H. TEMA: a software reference to shrimp *Litopenaeus vannamei* farming practices. Aquacult Econ Manag. 1999;3:267–80.

[36] De-qiang Z, Long-xing Y. Research on automatic control system of environmental factors in aquaculture. J Jiangsu Teach Univ Technol. 2006;12:34–7.

[37] Rao BS, Kameswari UJ. Monitoring system of aquiculture with automatic control system using ARM 7. Int J Comp Sci Infor Technol. 2012;3:3761–4.

[38] Ani DT, Cueto MGF, Diokno NJG, Perez KRR. Solar powered automatic shrimp feeding system. Asia Pac J Multidiscip Res. 2015;3:152–9.

[39] Appana DK, Alam MW, Basnet B. A novel design of feeder system for aquaculture suitable for shrimp farming. Int J Hybrid Infor Technol. 2016;9:199–212.

[40] Chandanapalli SB, Sreenivasa Reddy E, Rajya Lakshmi D. Design and deployment of aqua monitoring system using wireless sensor networks and IAR-Kick. J Aquac Res Dev. 2014;5:283.

[41] Mutalipassi M, Di Natale M, Mazzella V, Zupo V. Automated culture of aquatic model organisms: shrimp larvae husbandry for the needs of research and aquaculture. Animal. 2017;2:1–9. 10.1017/S1751731117000908 28462769

[42] Højris B, Christensen SC, Albrechtsen HJ, Smith C, Dahlqvist M. A novel, optical, online bacteria sensor for monitoring drinking water quality. Sci Rep. 2016;6:23935. 27040142 10.1038/srep23935 PMC4819223

[43] Hambly A, Stedmon C. FluoRAS sensor-online organic matter for optimising recirculating aquaculture systems. Res Ideas Outcomes. 2018;4:e23957.

[44] Parra L, Rocher J, Escrivá J, Lloret J. Design and development of low-cost smart turbidity sensor for water quality monitoring in fish farms. Aquac Eng. 2018;81:10–8.

[45] Parra L, Sendra S, García L, Lloret J. (2018) Design and deployment of low-cost sensors for monitoring the water quality and fish behavior in aquaculture tanks during the feeding process. Sensors. 2018;18:750. 29494560 10.3390/s18030750 PMC5877200

[46] Su X, Sutarlie L, Loh XJ. Sensors, biosensors, and analytical technologies for aquaculture water quality. Research (Wash DC). 2020;2020. 10.34133/2020/8272705. 10.34133/2020/8272705 PMC7048950 32149280

[47] Araneda M, Gasca-Leyva E, Vela MA, Domínguez-May R. Effects of temperature and stocking density on intensive culture of Pacific white shrimp in freshwater. J Therm Biol. 2020;94:102756. 33292997 10.1016/j.jtherbio.2020.102756

[48] Piérri V, Valter-Severino D, Goulart-de-Oliveira K, Manoel-do-Espírito-Santo C, Nascimento-Vieira F, Quadros-Seiffert W. Cultivation of marine shrimp in biofloc technology (BFT) system under different water alkalinities. Braz J Biol. 2015;75:558–64. 26292104 10.1590/1519-6984.16213

[49] Hlordzi V, Kuebutornye FKA, Afriyie G, Abarike ED, Lu Y, Chi S, Anokyewaa MA. The use of Bacillus species in maintenance of water quality in aquaculture: a review. Aquacult Rep. 2020;18:100503.

[50] Shi Y, Zhang G, Liu J, Zhu Y, Xu J. Performance of a constructed wetland in treating brackish wastewater from commercial recirculating and super-intensive shrimp growout systems. Bioresour Technol. 2011;102:9416–24. 21852127 10.1016/j.biortech.2011.07.058

[51] Alfiansah YR, Hassenrück C, Kunzmann A, Taslihan A, Harder J, Gärdes A. Bacterial abundance and community composition in pond water from shrimpa quaculture systems with different stocking densities. Front Microbiol. 2018;9:2457. 30405548 10.3389/fmicb.2018.02457 PMC6200860

[52] Van Duc L, Song B, Ito H, Hama T, Otani M, Kawagoshi Y. High growth potential and nitrogen removal performance of marine anammox bacteria in shrimp-aquaculture sediment. Chemosphere. 2018;196:69–77. 29291516 10.1016/j.chemosphere.2017.12.159

[53] Gao D, Liu M, Hou L, Derrick YFL, Wang W, Li X, Zeng A, Zheng Y, Han P, Yang Y, Yin G. Effects of shrimp-aquaculture reclamation on sediment nitrate dissimilatory reduction processes in a coastal wetland of southeastern China. Environ Pollut. 2019;255:113219. 31539849 10.1016/j.envpol.2019.113219

[54] Nair RR, Rangaswamy B, Sarojini BSI, Joseph V. Anaerobic ammonia-oxidizing bacteria in tropical bioaugmented zero water exchange aquaculture ponds. Environ Sci Pollut Res Int. 2020;27:10541–52. 31940146 10.1007/s11356-020-07663-1

[55] Towatana P, Voradaj C, Panapitukkul N. Changes in soil properties of abandoned shrimp ponds in southern Thailand. Environ Monit Assess. 2002;74:45–65. 11893160 10.1023/a:1013802704889

[56] Kruse J, Koch M, Khoi CM, Braun G, Sebesvari Z, Amelung W. Land use change from permanent rice to alternating rice-shrimp or permanent shrimp in the coastal Mekong Delta, Vietnam: changes in the nutrient status and binding forms. Sci Total Environ. 2020;703:134758. 31767321 10.1016/j.scitotenv.2019.134758

[57] Panigrahi A, Saranya C, Sundaram M, Vinoth Kannan SR, Das RR, Satish Kumar R, Rajesh P, Otta SK. Carbon: Nitrogen (C:N) ratio level variation influences microbial community of the system and growth as well as immunity of shrimp (*Litopenaeus vannamei*) in biofloc based culture system. Fish Shellfish Immunol. 2018;81:329–37. 30016684 10.1016/j.fsi.2018.07.035

[58] Ramos-Corella K, Martínez-Córdova LR, Enríquez-Ocaña LF, Miranda-Baeza A, López-Elías JA. Bio-filtration capacity, oxygen consumption and ammonium excretion of *Dosinia ponderosa* and *Chione gnidia* (Veneroida: Veneridae) from areas impacted and non-impacted by shrimp aquaculture effluents. Rev Biol Trop. 2014;62(3):969–76. 25412529

[59] Kathyayani SA, Poornima M, Sukumaran S, Nagavel A, Muralidhar M. Effect of ammonia stress on immune variables of Pacific white shrimp *Penaeus vannamei* under varying levels of pH and susceptibility to white spot syndrome virus. Ecotoxicol Environ Saf. 2019;184:109626. 31536848 10.1016/j.ecoenv.2019.109626

[60] Katayama T, Nagao N, Kasan NA, Khatoon H, Rahman NA, Takahashi K, Furuya K, Yamada Y, Wahid MEA, Jusoh M. Bioprospecting of indigenous marine microalgae with ammonium tolerance from aquaculture ponds for microalgae cultivation with ammonium-rich wastewaters. J Biotechnol. 2020;323:113–20. 32768414 10.1016/j.jbiotec.2020.08.001

[61] Liu F, Li S, Yu Y, Sun M, Xiang J, Li F. Effects of ammonia stress on the hemocytes of the Pacific white shrimp *Litopenaeus vannamei*. Chemosphere. 2020;239:124759. 31518920 10.1016/j.chemosphere.2019.124759

[62] Wei D, Zeng S, Hou D, Zhou R, Xing C, Deng X, Yu L, Wang H, Deng Z, Weng S, Huang Z, He J. Community diversity and abundance of ammonia-oxidizing archaea and bacteria in shrimp pond sediment at different culture stages. J Appl Microbiol. 2021;130:1442–55. 33021028 10.1111/jam.14846

[63] Ringø E, Van Doan H, Lee SH, Soltani M, Hoseinifar SH, Harikrishnan R, Song SK. Probiotics, lactic acid bacteria and bacilli: interesting supplementation for aquaculture. J Appl Microbiol. 2020;129:116–36. 32141152 10.1111/jam.14628

[64] Patel CN, Chellapandi P. Anaerobic digestion of cotton seed cake using developed mixed consortia. Electr J Environ Agricult Food Chem. 2009;8:333–44.

[65] Chellapandi P, Prabaharan D, Uma L. Evaluation of methanogenic activity of biogas plant slurry for monitoring codigestion of ossein factory wastes and cyanobacterial biomass. Appl Biochem Biotechnol. 2010;162(2):524–35. 19911119 10.1007/s12010-009-8834-2

[66] Chellapandi P, Uma L. Evaluation of methanogenic activity of biogas plant slurry on ossein factory wastes. J Environ Sci Eng. 2012;54:10–3. 23741852

[67] Holt CC, Bass D, Stentiford GD, van der Giezen M. Understanding the role of the shrimp gut microbiome in health and disease. J Invertebr Pathol. 2020;21:107387. 10.1016/j.jip.2020.107387 32330478

[68] Schar D, Klein EY, Laxminarayan R, Gilbert M, Van Boeckel TP. Global trends in antimicrobial use in aquaculture. Sci Rep. 2020;10:21878. 33318576 10.1038/s41598-020-78849-3 PMC7736322

[69] Thornber K, Verner-Jeffreys D, Hinchliffe S, Rahman MM, Bass D, Tyler CR. Evaluating antimicrobial resistance in the global shrimp industry. Rev Aquac. 2020;12:966–86. 32612676 10.1111/raq.12367 PMC7319481

[70] Wang Y, Cao Y, Feng G, Li X, Zhu L, Liu S, Coulter JA, Gao Q. Integrated soil-crop system management with organic fertilizer achieves sustainable high maize yield and nitrogen use efficiency in Northeast China based on an 11-year field study. Agronomy. 2020;10:1078.

